# The Structural and Optical Properties of 1,2,4-Triazolo[4,3-*a*]pyridine-3-amine

**DOI:** 10.3390/molecules27030721

**Published:** 2022-01-22

**Authors:** Lucyna Dymińska, Jerzy Hanuza, Jan Janczak, Maciej Ptak, Radosław Lisiecki

**Affiliations:** 1 Department of Bioorganic Chemistry, Wroclaw University of Economics and Business, Komandorska 118/120, 53-345 Wroclaw, Poland; 2 Institute of Low Temperature and Structure Research, Polish Academy of Sciences, Okólna 2, 50-422 Wroclaw, Poland; j.hanuza@int.pan.wroc.pl (J.H.); j.janczak@int.pan.woc.pl (J.J.); m.ptak@int.pan.wroc.pl (M.P.); r.lisiecki@int.pan.wroc.pl (R.L.)

**Keywords:** 1,2,4-triazolo[4,3-*a*]pyridin-3-amine, XRD studies, molecular structure, spectroscopic analysis

## Abstract

The structural and spectroscopic properties of a new triazolopyridine derivative (1,2,4-triazolo[4,3-*a*]pyridin-3-amine) are described in this paper. Its FTIR spectrum was recorded in the 100–4000 cm^−1^ range and its FT-Raman spectrum in the range 80–4000 cm^−1^. The molecular structure and vibrational spectra were analyzed using the B3LYP/6-311G(2d,2p) approach and the GAUSSIAN 16W program. The assignment of the observed bands to the respective normal modes was proposed on the basis of PED calculations. XRD studies revealed that the studied compound crystallizes in the centrosymmetric monoclinic space group *P*2_1_/*n* with eight molecules per unit cell. However, the asymmetric unit contains two 1,2,4-triazolo[4,3-*a*]pyridin-3-amine molecules linked via N–H⋯N hydrogen bonds with a R_2_^2^(8) graph. The stability of the studied molecule was considered using NBO analysis. Electron absorption and the luminescence spectra were measured and discussed in terms of the calculated singlet, triplet, HOMO and LUMO electron energies. The Stokes shifts derived from the optical spectra were equal to 9410 cm^−1^ for the triazole ring and 7625 cm^−1^ for the pyridine ring.

## 1. Introduction

Triazolopyridine derivatives constitute an important class of organic compounds employed in various biochemical, clinical and pharmaceutical applications. This group includes [1,2,4]triazolo[1,5-*a*]pyridine, [1,2,4]triazolo[4,3-*a*]pyridine, [1,2,3]triazolo [1,5-*a*]pyridine, [1,2,3]triazolo[4,5-*b*]pyridine and [1,2,3]triazolo[4,5-*c*]pyridine isomeric systems exhibiting particularly useful effects. They have been recognized as antifungal [1,2,3,4], insecticidal [4], antibacterial [5,6], anticonvulsant [7], antioxidant [8], herbicidal [9] agents, as well as adenosine receptors [10] or HIF prolyl hydrolase [11] and myeloperoxidase [12] inhibitors. On the other hand, pyridotriazine derivatives were discovered as prospective drugs showing antithrombotic [13], antimicrobial [14,15], antifungal [16] and anti-Alzheimer disease [17] effects. They are also antagonists of serotonin receptors [18,19].

Several isomeric triazolopyridines are of peculiar importance in medicinal and pharmaceutical applications. Special efforts have mainly been focused on [1,2,4]triazolo[4,3-*a*]pyridine, which is recognized as a member of a new class of antidepressants that chemically and pharmaceutically differ from the other tricyclic drugs [1,2,3,4,5,6,7,8,9,11,15]. Trazodone, an effective antidepressant of the serotonin antagonist showing anxiolytic and hypnotic effects [20,21,22], is an example of their application. Its transfer into breast milk and excretion was carefully studied in [23].

[1,2,3]triazolo[4,5-*b*]pyridine derivatives offer somewhat limited application possibilities. They have been described as anti-inflammatory, hypotensive, hypoglycemic, antipyretic, analgesic, antiasthmatic drugs and as vasodilators and substrates of NAD glycohydrolase [24,25,26,27,28].

[1,2,3]triazolo[4,5-*c*]pyridine was reported to be an organic sensitizer for high-performance solar cells [29,30] due to the formation of a D-A-π-A organic core where D is the electron donor, A is the electron acceptor and π is the conjugated spacer between them. Yutilov and Smolyar [31] described its synthesis.

Finally, the syntheses of several [1,2,4]triazolo[1,5-*a*]pyridine derivatives have been reported, for which the prospective medical and pharmaceutical applications were suggested [10].

A very important contribution of the triazolopyridines (TPs) to the medical and pharmaceutical fields is related to receptor–ligand interactions and their docking to biologically active components. The docking strategy was used for 1,2,4-TP as a 11β-HSD1 inhibitor [32], histone demethylase activator [33], antagonists for the adenosine receptor [34,35] or antiproliferative inhibitors of tubulin polymerization [36]. It was discovered that 7-aryl-1,2,4–TPs react with glutamate receptors leading to new drugs active in psychiatric diseases (schizophrenia, anxiety disorder) [37].

The nitrogen-containing heterocyclic compounds such as triazolopyridines often occur as bi- or three-dentate chelating ligands due to the presence of the lone electron pairs on the nitrogen atoms. They can coordinate metal ions and form complex compounds applied as fluorescence sensors, chemisensors and catalysts [38,39,40,41,42,43,44,45]. The aim of the present work was to characterize the structural and spectroscopic properties of a new compound, 1,2,4-triazolo[4,3-*a*]pyridin-3-amine, in the solid-state. Spectral measurements and quantum chemical calculations were undertaken to determine the energies and conformation of the double triazolopyridine ring. Besides, because such a double-ring system can act as a donor to a metal center, a phosphorescence sensor or a chemisensor, the knowledge of its optical and electron properties is important for the full characterization of the studied compound. These data can determine whether this material can serve as a photochromic system. A full structural, electronic and optical characterization of the triazolopyridine derivatives is important in understanding their receptor–ligand interactions and docking processes in the production of the drugs [35].

## 2. Materials and Methods

**Figure 1:**
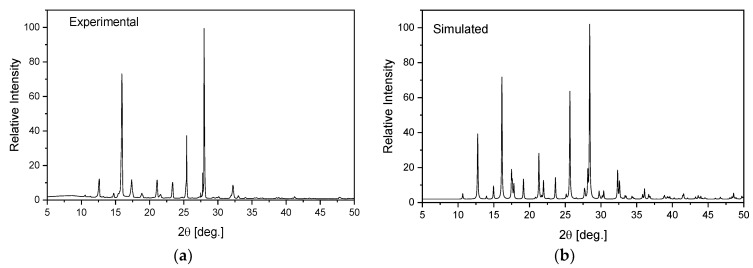
Experimental (**a**) and simulated (**b**) from an X-ray structural analysis on a single crystal for TPa-NH_2_.

1,2,4-triazolo[4,3-*a*]pyridin-3-amine (Sigma Ltd., Rowville, Australia) was recrystallized from an ethanol solution. The obtained orange single crystals were studied using the XRD technique. X-ray intensity data for the title crystal were collected using graphite monochromatic MoKα radiation on a four-circle κ geometry Xcalibur diffractometer with Sapphire2 area CCD detector at 100 K. Data collections were carried out using the CrysAlis CCD program [46]. Integration, scaling of the reflections, correction for Lorentz and the polarisation effects and absorption corrections were performed using the CrysAlis Red program [46]. The structures were solved by the direct methods using SHELXT-2014/7 [47] and refined using the SHELXL-2018/3 program [48]. The positions of the hydrogen atoms in the pyridine ring were introduced in their geometrical positions and treated as rigid, whereas the H atoms of the NH_2_ group were refined. The final difference Fourier maps showed no peaks of chemical significance. Details of the data collection parameters, crystallographic data and final agreement parameters are presented in Table S1. The selected geometrical parameters are shown in Table 1 and the geometry of the closest intermolecular interactions is exhibited in Table 2. Visualizations of the structures were made with the Diamond 3.0 program [49]. The structure in the CIF format was deposited in the Cambridge Crystallographic Data Centre (CCDC No. 2115902). The homogeneity of the obtained sample was checked on a PANanalytical X’Pert diffractometer equipped with a Cu-Kα radiation source (λ = 1.54182 Å). The diffraction data were recorded in the range of 5–50° at room temperature. The experimental pattern of powder diffraction was quite similar to that simulated from the X-ray single crystal structure and indicates the homogeneity of the obtained sample (Figure 1).

The FT-IR/ATR spectrum was recorded in the 300–4000 cm^−1^ range using a Nicolet 6700 spectrometer (Thermo Fisher Scientific, Waltham, USA) equipped with a portable ATR set. The resolution of these measurements was 2.0 cm^−1^.

The FT-Raman spectrum in the 4000–80 cm^−1^ range was measured in back scattering geometry with a FT Bruker 110/S spectrometer. The resolution was 2.0 cm^−1^. The YAG:Nd laser was used as an excitation source (excitation wavelength 1064 nm).

The room temperature electron absorption spectrum was measured in the 200–1500 nm spectral range using a Cary-Varian 5E UV-VIS-near-IR spectrophotometer with a resolution of 0.5 nm. The absorption spectrum of the compound in the ground state was recorded in silicon paste.

Emission spectra and luminescence decay curves were recorded with a grating spectrograph (Princeton Instr. Acton, Massachusetts 01720 USA, Model Acton 2500i) coupled to a streak camera (Hamamatsu, 314-5, Shimokanzo, Iwata City, Shizouka Pref., 438-0193, Japan, Model C5680) operating in the 200–1100 nm spectral range with a temporal resolution of 20–100 ps. The luminescent properties of the compounds were investigated at room temperature.

The geometry optimization of the molecular structure of the studied compound was carried out for the monomer and dimer with the use of the GAUSSIAN 16W program package [50]. All calculations were carried out using density functional three-parameters hybrid (B3LYP) methods [51,52,53] with the 6-311G(2d,2p) approach [54,55,56,57]. The calculated and experimental values were compared using scaling factors to correct the evaluated wavenumbers for vibrational anharmonicity and deficiencies inherent to the used computational level. The scaling factors were determined using the mean square method [58,59]. The Potential Energy Distribution (PED) of the normal modes among the respective internal coordinates was calculated for the studied derivatives using the BALGA [60] program.

The natural bond orbitals calculations were carried out using the NBO program as implemented in the GAUSSIAN 16W package at the B3LYP/6-311G(2p,2d) level of theory in order to show a detailed description of the electronic structure of the TPa-NH_2_ molecule.

## 3. Results and Discussion

### 3.1. Crystal and Molecular Structure

**Figure 2:**
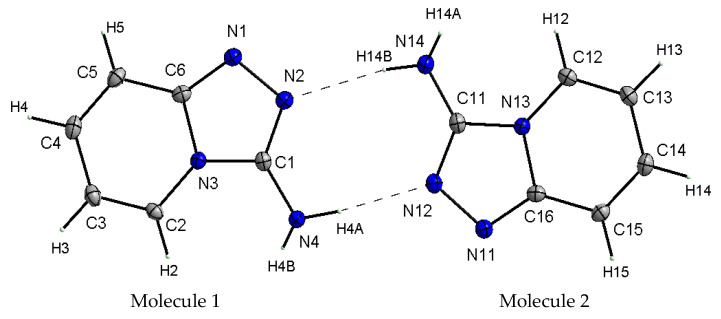
View of the asymmetric unit of TPa-NH_2_ with the labeling scheme. Displacement ellipsoids are shown at the 50% probability level, H atoms with arbitrary radii.

X-ray studies of the TPa-NH_2_ single crystal showed that the studied compound crystallizes in the monoclinic system in the centrosymmetric space group *P*2_1_/*n* with eight molecules per unit cell. Its unit cell parameters are a = 5.5666 Å, b = 12.6649 Å, c = 16.8190 Å and β = 99.434^0^. However, the asymmetric unit contains two 1,2,4-triazolo[4,3-*a*]pyridin-3-amine molecules linked via N–H⋯N hydrogen bonds with a R_2_^2^(8) graph (Figure 2). It should be noted that the center of the R_2_^2^(8) system is not the inversion crystallographic center. Details of the data collection parameters, crystallographic data and final agreement parameters are presented in Table S1.

**Figure 3:**
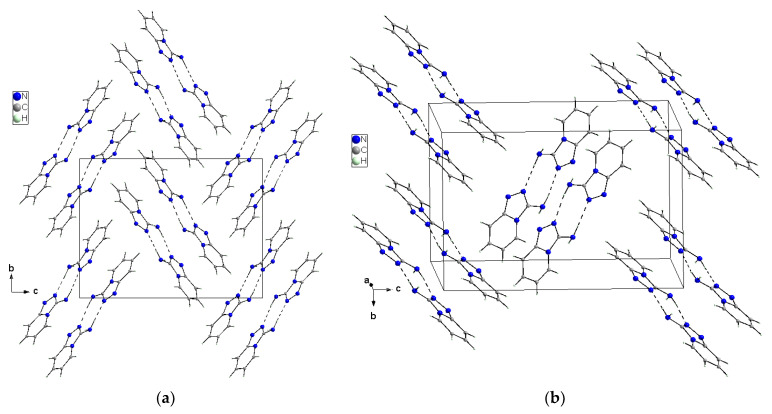
Crystal packing of the TPa-NH_2_ molecules in the unit cell showing the N–H⋯N hydrogen-bonded dimers with an R_2_^2^(8) graph viewed almost along [100] (**a**) and [210] directions (**b**).

X-ray selected geometrical parameters with DFT-optimized settings are presented in Table 1 and the geometry of the hydrogen bonding interactions is shown in Table 2. The respective bond lengths in both the independent molecules obtained from the X-ray analysis of single crystals are almost the same and correlate nicely with the bond lengths of the gaseous molecule obtained from the DFT calculations. The conformations of both the independent molecules exhibit a similar, almost planar conformation. The dihedral angle between the plane of the pyridine ring and the plane of the 1,2,4-triaza ring is equal to 3.1(1)° in molecule 1 and 1.8(1)° in molecule 2. However, small differences between the X-ray and DFT values of the studied compound are due to different approaches to the description of the conformation of the molecules. The X-ray values refer to the conformation of the molecules in crystals where the interactions between the molecules play a significant role and lead to crystallization and a specific crystal packing, while DFT values refer to a single isolated molecule in the gaseous state, omitting interactions between molecules and thus leading to these differences. The arrangement of the molecules in crystal is mainly determined by the N–H⋯N hydrogen bonds that form a dimeric structure with a R_2_^2^(8) graph and the dimers mainly interact in the unit cell by electrostatic interactions, dispersive forces and van der Waals forces because there are no strong directional interactions (such as hydrogen bonds) between the dimers (Figure 3). The dimers are arranged in stacks along and perpendicular to the [210] direction (see Figure 3b). Within the stacks, the π⋯π interaction between the clouds of the rings is relatively weak due to the distance of 3.710(5)Å between Cg⋯Cg (Cg = gravity center of the pyridine ring). The geometry of the hydrogen bonds and the other closest contacts between the molecules are presented in Table 2.

**Figure 4:**
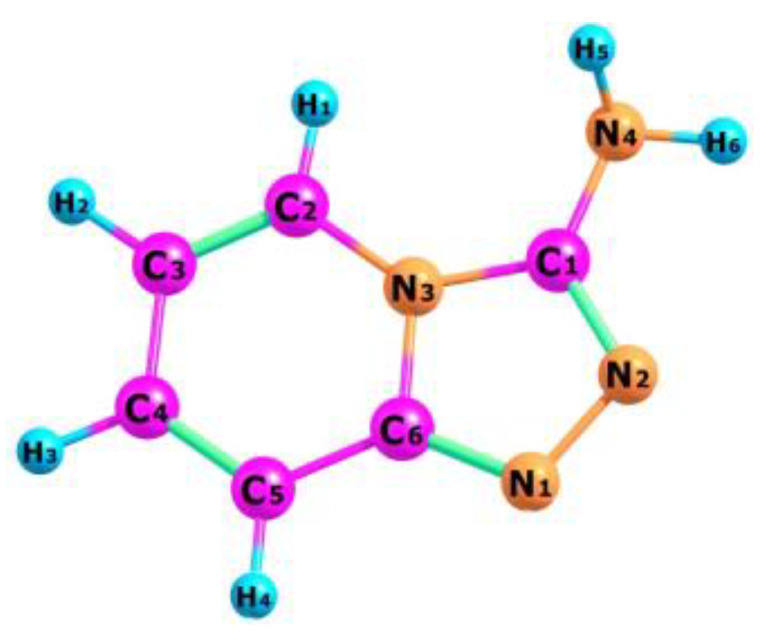
Molecular structure of TPa-NH_2_ with numbering atoms.

The optimized structure of the TPa-NH_2_ molecule obtained from the DFT calculations is shown in Figure 4, in which the numbering of the atoms is also defined. The calculated geometrical parameters are introduced in Table 1 for comparison with those obtained from the XRD studies. These values were used to calculate the vibrational and electron energy levels. It should be noted that the theoretical and experimental bond lengths, the angles between them as well as the torsional angles show a very good accordance which proves that the theoretical model of the DFT calculations was chosen correctly.

The Mulliken atomic charges determined in the present work for the studied compound are presented in Table S2. These results show that coupling between the pyridine and triazole rings as well as the substitution of the amino group at C(1) atom leads to a redistribution of electron density. The charges of the nitrogen atoms clearly vary depending on their place and function in the TP system. The N(1) and N(2) atoms in the triazole ring exhibit charge values of −0.279 and −0.281, respectively. The third nitrogen N(3) atom exhibits a higher charge value equal to −0.243 because it is common for the both rings. The charge value of the fourth N(4) atom is significantly smaller (−0.406) because it belongs to the amino-chromophore engaged in the hydrogen bond joining two TP molecules in the unit cell. The charges of the carbon atoms also depend on their position in the TP system. The pyridine carbons are symmetrically (negative or positive) distributed in this ring: C(2): 0.118, C(3): −0.183, C(4): 0.044, C(5): −0.163. As is common for T and P rings, the C(6) atom has a significantly higher charge value: 0.372. A clearly higher value (0.420) characterizing the charge of C(1) atom is due to the attachment of an amino group in this place.

The charge values of the hydrogen atoms attached to the pyridine ring are nearly similar: 0.0676, 0.063, 0.068 and 0.084. They clearly differ from those of the hydrogen atoms of the amino group: 0.152 and 0.166—one of them is engaged in the inter-molecular hydrogen bond.

Because the amino-chromophore, triazole nitrogen atoms and hydrogen atoms participate in the HB interactions between the TPa-NH_2_ units of the dimer, the charge distribution in this system reflects the changes resulting from the dimer formation. The following Mulliken’s charges were fixed for amino-chromophore nitrogen N(4), triazole nitrogen N(2) and the hydrogen atom H(6) of HB for the dimer: −0.405; −0.327; and 0.188, respectively. This means that the formation of the dimer causes the charge increase in the bridging hydrogen and a charge decrease in the triazole nitrogen.

Table S3 presents the values of the stabilization energies E(2) associated with the hyper conjugative interactions within the molecule.

The stabilization energy of the molecule is described by the difference between the energy of a delocalized chemical structure and the theoretical energy of a structure with localized bonds. It reflects the interactions between the bonds inside the molecule as the measure of the stability gained when two atoms bond with each other, as opposed to their free or unbound states. These parameters are particularly useful for the aromatic molecules in which π interactions couple the whole system. Such a situation appears for the studied TP system in which N2=C1, N1=C6, C4=C5 and C2=C3 bonds exhibit a π character. The following energy values are particularly expressive: π(N2–C1)–π*(N1–C6) = 12.04 kcal/mol, π(N1–C2)–π*(N2–C1) = 14.79, π(C4–C5)–π*(N1–C5) = 21.77, π(C4–C5)–π*(C2–C3) = 16.42 and π(C2–C3)–π*(C4–C5) = 14.85 kcal/mol. They express the π interactions between the double bonds in the TP system shown in Figure 2 and Figure 4 and structural parameters from Table 1.

### 3.2. FTIR and Raman Spectra

**Figure 5:**
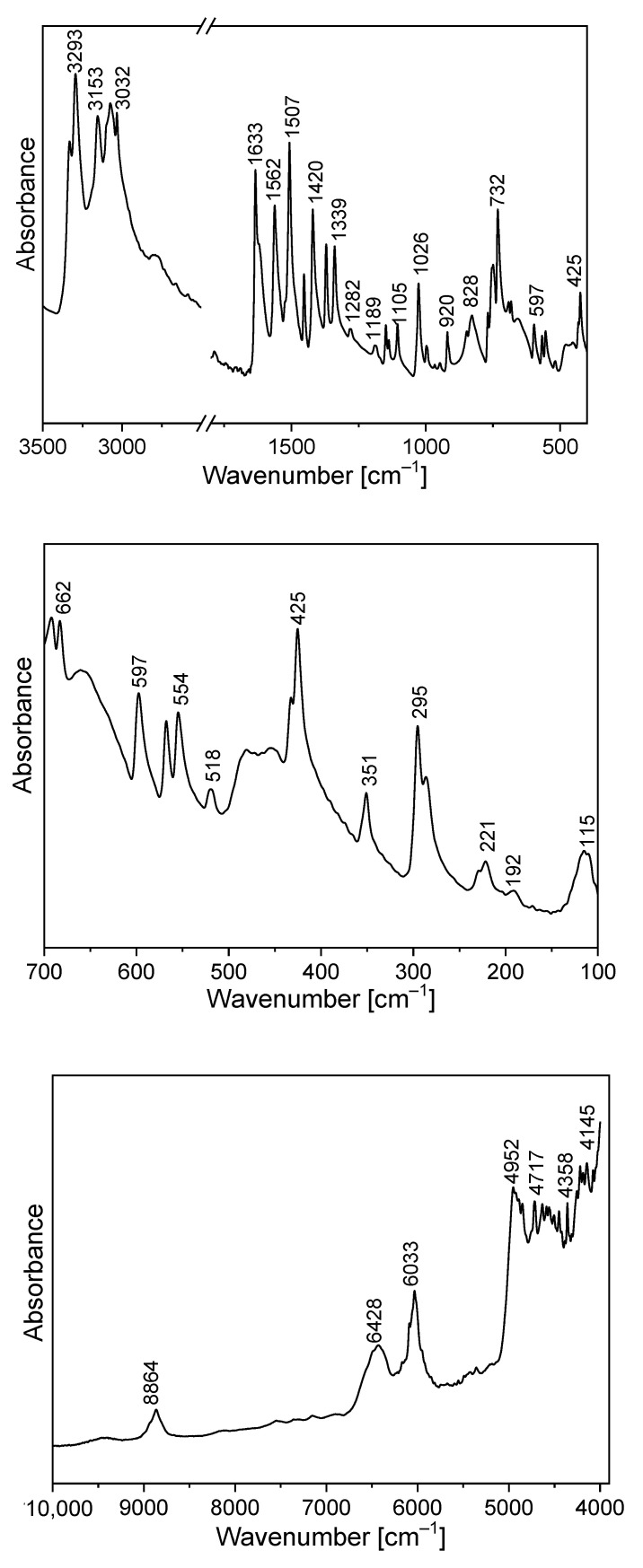
Infrared spectrum of TPa-NH_2_.

**Figure 6:**
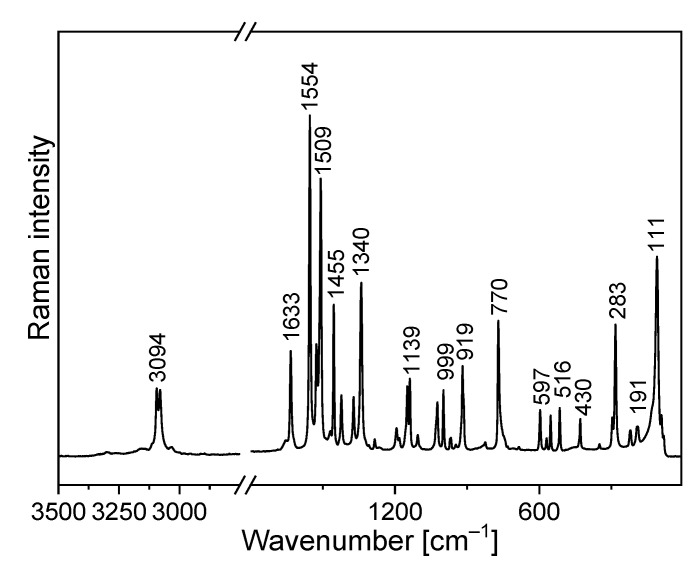
Raman spectrum of TPa-NH_2_.

**Figure 7:**
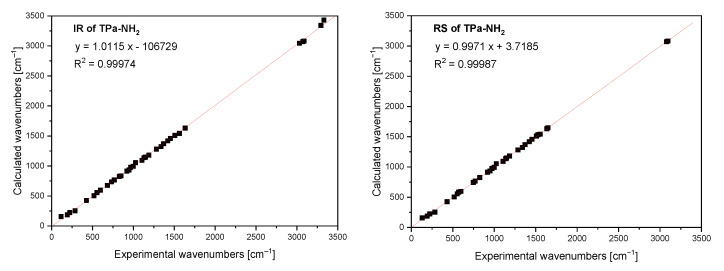
Comparison of the experimental and calculated infrared and Raman spectra of TPa-NH_2_.

The FTIR and Raman spectra of the studied compound are shown in Figure 5 and Figure 6. Table S4 presents the observed band wavenumbers and their assignment to the respective vibrational modes. The correlation graphs of the experimental and calculated wavenumbers are presented in Figure 7, showing their good agreement.

The amino-triazolopyridine molecule contains two clearly distinguishable vibrational patterns corresponding to the triazolopyridine skeleton and the amino group bonded to these units. The nine-atomic triazolopyridine skeleton gives rise to twenty-one vibrational normal modes. The theoretical calculations and vibrational spectra show that these normal modes can be subdivided into those in which one ring vibrates only, i.e., pyridine or triazole rings separately and in which the concerted motions of the whole triazolopyridine double-ring system participates. The vibrations of two former units are characterized by in-plane stretching ν(φ_P_) vibrations; in-plane δ(φ_P_) bending vibrations; out-of-plane bending γ(φ_P_) vibrations; stretching ν(φ_T_); in-plane bending δ(φ_T_); and out-of-plane bending γ(φ_T_). On the other hand, the concerted vibrations of the whole triazolopyridine skeleton (Φ) are described by the modes that could be denoted as stretching ν_as_(Φ), ν_s_(Φ), γ(Φ) wagging and τ(Φ) wagging, because the whole double ring Φ system participates in these vibrations. The wavenumbers of ν_as_(Φ) modes were calculated at 1324 cm^−^^1^ for the monomer and 1331 cm^−^^1^ for the dimer. In the IR spectrum, this mode was observed at about 1339 cm^−^^1^ and in the Raman spectrum—at 1340 cm^−^^1^. The second characteristic in-plane bending vibration of the triazolopyridine skeleton is described by the mode calculated at 767 cm^−^^1^ for the monomer and at 777–778 cm^−^^1^ for the dimer. The ν_s_(Φ) vibration was observed in the 766−770 cm^−^^1^ range. Its intensity in the Raman spectrum is significantly higher than its IR counterpart. The mode calculated at 426 cm^−^^1^ for the monomer and 425–427 cm^−^^1^ for the dimer should be denoted as the γ(Φ) wagging motion of the entire skeleton. This mode was observed at 425 cm^−^^1^ in the IR spectrum and 430 cm^−^^1^ in the Raman spectrum. The τ(Φ) wagging modes calculated at about 188 cm^−^^1^ for the monomer and 196–203 cm^−^^1^ for the dimer were observed in the IR spectrum at 192 cm^−^^1^ and in the Raman spectrum at 191 cm^−^^1^.

The same modes of the double triazolopyridine rings were described in our previous works for 7-methyl-1*H*-[1,2,3]triazolo[4,5-*c*]pyridinium nitrate (7MTPHc) and 6-methyl-1*H*-[1,2,3]triazolo[4,5-*b*]pyridine (6MTPb) which contain triazolo[4,5-*c*]- and triazolo[4,5-*b*]-pyridine skeletons in their structure [61,62]. The wavenumbers corresponding to the characteristic vibrations of triazolo[4,5-*b*]-, triazolo[4,5-*c*]- and triazolo[4,3-*a*]pyridine skeletons are shown in Table 3.

Comparing the theoretical and experimental wavenumbers, it should be noted that the amino group participates in intermolecular interactions in the unit cell. N−H⋯N hydrogen bonds (HBs) play an important role in chemistry and biology. Such interactions are expected for the TPa derivatives. The XRD data postulate the formation of the N−H⋯N HB. For the solid state in which such interactions appear, the N−H⋯N hydrogen bond is formed between the adjacent TPa-NH_2_ units of the unit cell. A HB is formed between the amino chromophore of one molecule and the nitrogen acceptor of the adjacent unit’s triazole ring.

The theoretical calculations for TPa-NH_2_ were carried out for both the monomer and the dimer. The characteristic vibrations of the HB for the dimer were predicted by DFT calculations at the following wavenumbers: ν(N−H⋯N) 3126 and 3096 cm^−1^; δ(N−H⋯N) at 1428 and 1426 cm^−1^; γ(N−H⋯N) at 884 and 870 cm^−1^; and ν(N−H)⋯N at 104 cm^−1^.

The FTIR spectrum of TPa-NH_2_ exhibits a very broad and strong bond in the range 2700–3400 cm^−1^. The ν(N−H⋯N) vibration was observed at 3153 cm^−1^. The band at 1420 cm^−1^ of the IR spectrum should be assigned to the δ(N–H⋯N) in-plane bending vibrations. This vibration was observed in the Raman spectrum at 1423 cm^−1^. The characteristic band in the range 800–900 cm^−1^ of the FTIR spectra should be assigned to the out-of-plane bending vibrations γ (N–H⋯N).

The NIR spectrum (Figure 5) of the studied derivative contains a rich spectral pattern of overtones and combination modes. They can be assigned to the following mixed components: 9460 cm^−1^: 3ν_3_; 8864 cm^−1^: 2ν_1_ + 2ν_20_ or 2ν_3_ + 2ν_16_; 7500 cm^−1^: 2ν_1_ + ν_26_; 6428 cm^−1^: ν_1_ + 2ν_9_; 6033 cm^−1^: 2ν_6_; 5357 cm^−1^: ν_2_ + ν_8_ + ν_37_; 4952 cm^−1^: 3ν_7_; 4717 cm^−1^: ν_2_ + ν_13_ or ν_3_ + ν_9_; 4570 cm^−1^: ν_2_ + ν_16_ or 3ν_10_; 4358 cm^−1^: 3ν_12_ or ν_1_ + ν_21_ or ν_4_ + ν_16_; 4145 cm^−1^: ν_1_ + ν_27_ or ν_2_ + ν_26_ or ν_3_ + ν_22_. The discrepancies between the wavenumbers observed in the NIR region, corresponding to combinations of fundamental transitions and theoretical values, do not exceed a few cm^−1^ which means that the scaling factors used in the DFT calculations were taken properly.

### 3.3. Electron Absorption and Emission Spectra

**Figure 8:**
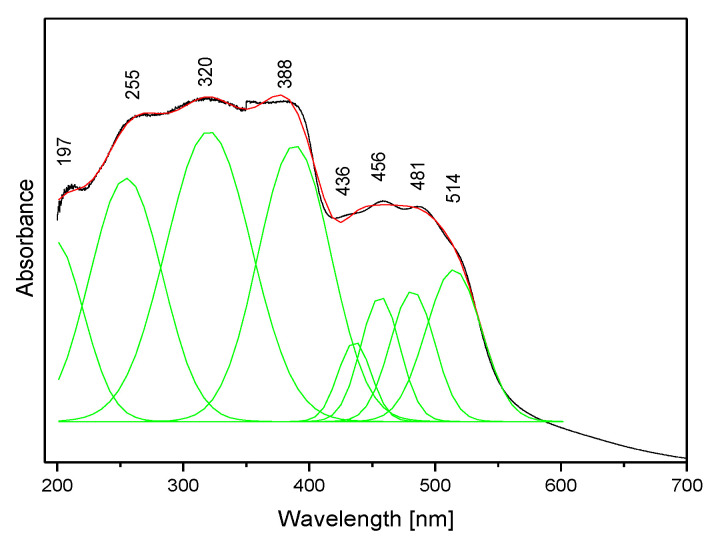
The electron absorption spectrum of the TPa-NH_2_ molecule.

The absorption spectrum of the studied compound is shown in Figure 8. It contains two clear bands: the stronger complex spectral pattern in the range 200–400 nm and the weaker doublet in the range 400–600 nm. Such a spectral pattern agrees with those reported for other triazolopyridines [63,64] and the fact that the title molecule contains two coupled π-ring systems.

To learn about the electronic properties of the title compound, DFT calculations were carried out using the optimized geometry in this molecule. Knowledge of the singlet and triplet energy levels of the ligand is needed to assign the observed bands to the respective electron transitions. The distribution of these levels was determined by the quantum chemical DFT approach. The results of these evaluations are shown in Table 4. The singlet states of the studied derivative fall in the range 188–327 nm, but the energies of the triplet levels range from 211 to 479 nm. These data could be compared with the experimental values seen in the electron absorption spectrum presented in Figure 8.

**Figure 9:**
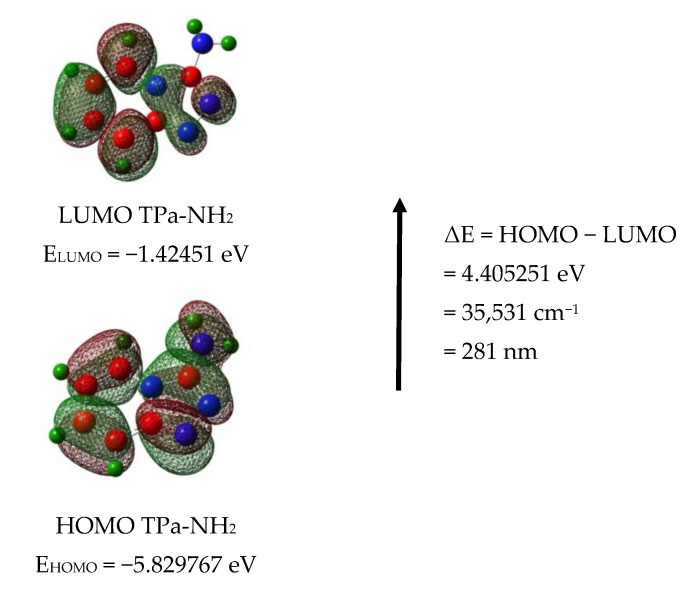

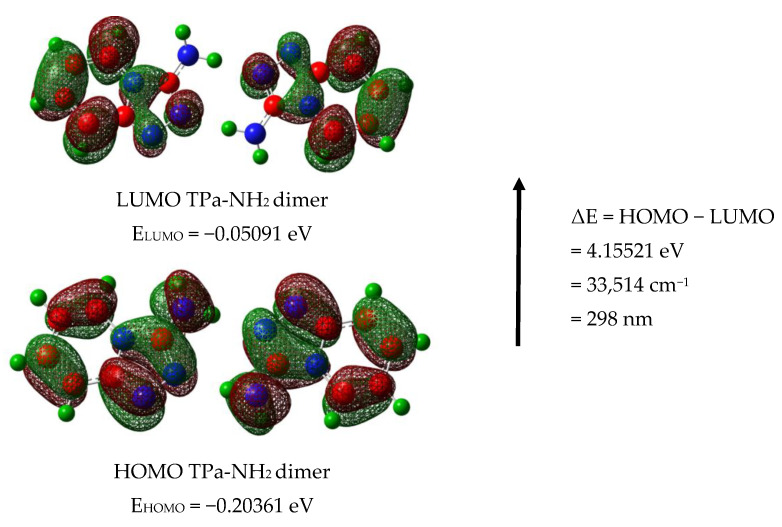
HOMO and LUMO molecular orbitals for the TPa-NH_2_ monomer and dimer.

The calculated energies of the singlet states show a good agreement with the position of the observed bands (Figure 8). The calculated oscillator strengths of the transitions at 212 and 204 and 217 nm (No. 6 and 7) have the greatest values which fit well with the strong bands observed in the range 200–250 nm. The energy of the band at 321 nm coincides with that of the singlet state No. 1 for which the calculated value equals 326 nm. It is difficult to assign the bands observed at 456 and 500 nm. They could be explained as a result of the intermolecular interaction between the TP molecules in the unit cell. The presence of the NH_2_ group in the title molecule provides additional possibilities for the intermolecular charge transfer by H-bonding, π-π* stacking or donor–acceptor interactions [65]. Such a mechanism follows from the scheme of the HOMO → LUMO transition presented in Figure 9. ΔE for these levels is equal to 281 nm (35,531 cm^−1^). This corresponds to the band observed in the UV-Vis spectrum at about 255 nm. HOMO orbitals are localized both on the rings and the amino group, but the LUMO orbitals are reduced mainly to the three nitrogen atoms of the whole ring system with a small contribution of the C–H bonds.

Taking into account the theoretical and experimental data, the observed bands should be assigned to the following transitions: 200–289 nm corresponds to the π → π* of the triazole and pyridine rings and above 300 nm to the n → π* of this system. The energy of the HOMO → LUMO transition suggests that the lowest singlet state assigned to the π → π* intramolecular charge transfer (ICT) can overlap with some contribution to the n → π* (CT) transition.

**Figure 10:**
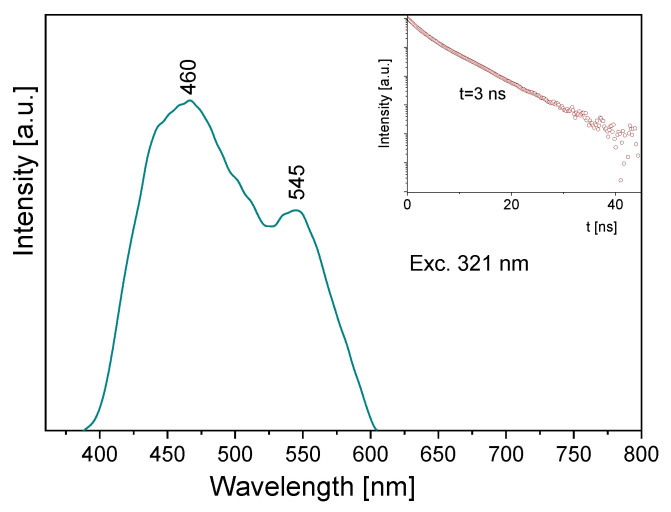
The emission spectrum of the TPa-NH_2_ molecule. Inserts show the emission lifetime for the TPa-NH_2_ molecule excited at 321 nm.

Some information follows from the luminescence spectrum measured for the title compound (Figure 10). Its emission spectrum contains two bands: a high intensity one at about 460 nm and the second, clearly weaker, at 545 nm. Depending on the wavelength used for the excitation line, the former band is somewhat shifted towards blue. The observed bands should be assigned to the π* → n transitions of the pyridine and triazole rings, respectively. The lifetime of the excited state is 3 ns (insert of Figure 10). A comparison of the absorption and emission spectra allows us to state that the Stokes shift for the measured sample is close to 9410 and 7625 cm^−1^, respectively for the triazole and pyridine rings.

**Figure 11:**
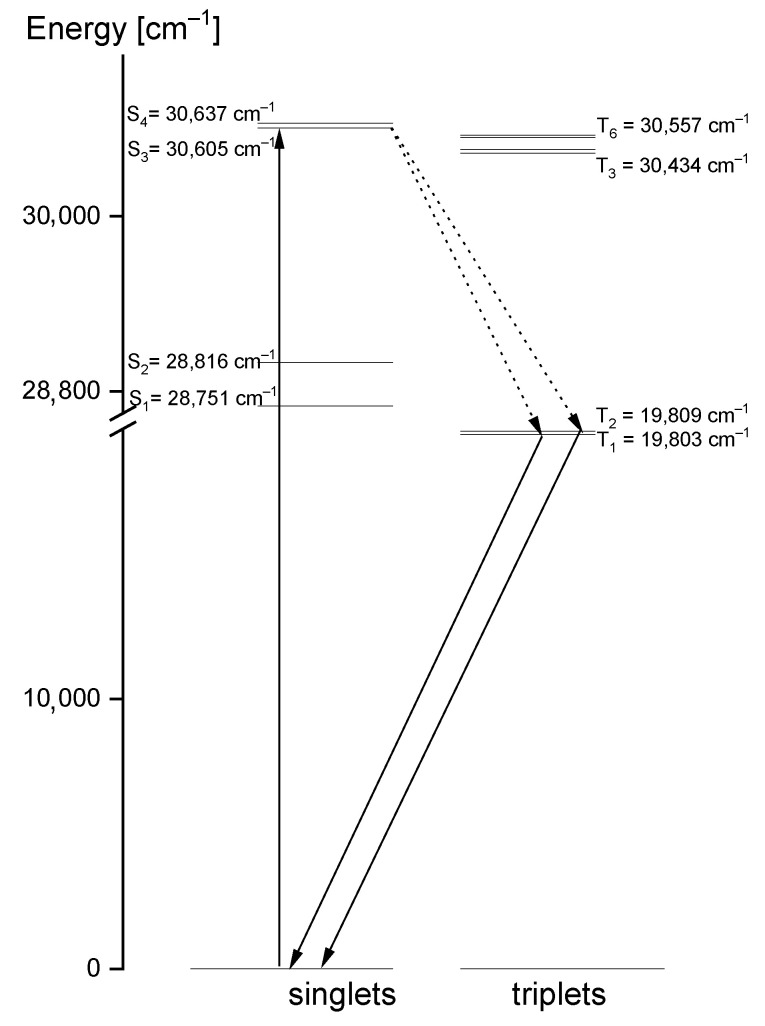
Depopulation mechanism of the excited states proposed for the studied 1,2,4-triazolo[4,3-*a*]pyridin-3-amine.

Comparing the theoretical data with the UV-Vis and emission spectra of the studied compound, another explanation of its electron properties can be proposed. The luminescence of the title compound was observed in the range 460–545 nm. It clearly shifted into red in relation to the S_0_ → S_1_ absorption transition, calculated at 326 nm and observed at 320 nm. Therefore, the observed luminescence could originate from the T_1_,T_2_ → S_0_ emission that may have proceeded by the inter-system crossing S_1_ → T_1_,T_2_. Room temperature fluorescence is generally not observed from excited triplet states; however, having in mind the calculated energies of these levels, we believe that a transition of this character is reasonable. According to the DFT calculations, the energies of the T_1_ and T_2_ states were 20,896 cm^−1^ and 31,272 cm^−1^, respectively, i.e., they were close to those expected for the emission from the triplet state. Besides, their red shift agrees with the expectations because the inter-molecular hydrogen bonds significantly affect the excited state properties. The excited-state hydrogen bonding influences nonadiabatic processes such as internal conversion, intersystem crossing, intramolecular charge transfer and photoinduced electron transfer. Giving such an explanation of the discrepancies between the theoretical and experimental data, the depopulation scheme of the excited states for the studied compound can be proposed (Figure 11). It is based on the intersystem crossing mechanism which is a part of a Jablonsky diagram often used to explain the origins of the luminescence observed for organic compounds [66,67,68,69,70]. This scheme clearly explains why in the emission spectrum there is a doublet band: it results from the appearance of the T_1_ → S_0_ and T_2_ → S_0_ transitions.

## 4. Conclusions

1. 1,2,4-triazolo[4,3-*a*]pyridin-3-amine occurs in a monoclinic structure forming the orange crystals—eight molecules constitute its unit cell. Its full structure has been described in the present work for the first time.
2. IR and Raman spectra measurements confirm the existence of inter-molecular N–H⋯N hydrogen bonds in which the NH_2_ group attached to the triazole ring joins the triazole nitrogen atom of the adjacent TP system. The amino group plays the role of an effective hydrogen bond donor.
3. A characteristic set of normal modes was identified for the triazolopyridine skeleton in the title molecule and their PED contributions were characterized (see Table S4).
4. The TD-DFT B3LYP quantum–chemical calculations performed for the isolated TPa-NH_2_ molecule located the lowest singlet state assigned to the π → π* intramolecular charge transfer (ICT) transition that probably overlaps with some contribution to the n → π* (CT) transition.
5. The presence of an amino group in 1,2,4-triazolo[4,3-*a*]pyridin-3-amine, its electron properties and the energies of its ground and excited states suggest that this compound can be used as a ligand for the complexation of the d- and f- electron ions. The hydrogen atom of the amino group participating in the intermolecular hydrogen interaction is susceptible for the complexing of the metal ions. In such a binding, the lone pairs of nitrogen atoms of the triazole ring can participate. Besides, a very broad range of the electron absorption in the studied compound allows them to become excited in metal complexes through the ligand-to-metal transfer. The probable depopulation mechanism of the excited states of the ligand is proposed in the present work (Figure 11).

## Figures and Tables

**Table 1:** X-ray and DFT optimized geometrical parameters (Å,°) of TPa-NH_2_.

X-ray Values			DFT
Bonds	Molecule 1	Molecule 2 *	
N1—N2	1.4005 (11)	1.4024 (11)	1.374
N2—C1	1.3147 (13)	1.3164 (13)	1.311
C1—N3	1.3760 (12)	1.3739 (12)	1.370
N3—C2	1.3827 (12)	1.3865 (12)	1.376
C2—C3	1.3507 (13)	1.3536 (13)	1.355
C3—C4	1.4337 (14)	1.4330 (14)	1.431
C4—C5	1.3601 (14)	1.3599 (14)	1.363
C5—C6	1.4281 (13)	1.4283 (13)	1.416
C6—N3	1.3863 (12)	1.3896 (12)	1.401
C6—N1	1.3211 (13)	1.3182 (12)	1.317
C1—N4	1.3716 (12)	1.3672 (13)	1.393
N4—H4A	0.912 (14)	0.900 (15)	1.014
N4—H4B	0.884 (14)	0.884 (15)	1.012
**Angles**			
N1—N2—C1	108.46 (8)	108.56 (8)	108.83
N2—C1—N3	109.53 (8)	109.56 (8)	110.12
C1—N3—C6	105.30 (8)	105.11 (8)	104.04
C1—N3—C2	131.42 (8)	131.38 (8)	132.96
C2—C3—C4	120.54 (9)	120.69 (9)	120.49
C3—C4—C5	121.11 (9)	121.33 (9)	120.90
C4—C5—C6	118.47 (9)	118.46 (9)	119.04
C5—C6—N1	131.73 (9)	131.51 (9)	119.03
C5—C6—N3	118.26 (8)	118.13 (8)	118.05
N3—C6—N1	110.02 (8)	110.34 (8)	109.89
N2—C1—N4	128.31 (9)	127.91 (9)	127.39
H4A—N4—H4B	113.8 (12)	115.3 (13)	108.69
**Torsion Angles**			
N1—N2—C1—N4	−177.34 (9)	177.52 (9)	−175.56
N1—C6—C5—C4	−176.21 (10)	−179.85 (10)	−178.91
N2—N1—C6—C5	−178.68 (10)	177.61 (10)	−178.87
N2—C1—N3—C2	−175.45 (9)	178.12 (9)	−177.92
C1—N3—C2—C3	178.26 (9)	178.55 (9)	−179.76

* for Molecule 2, the description of atoms is preceded by the number 1 and the rest are the same as in Molecule 1 (see Figure 2).

**Table 2:** Hydrogen bond geometry (Å,°).

D—H⋯A	D—H	H⋯A	D⋯A	D—H⋯A
N4—H4A⋯N12	0.912 (14)	2.088 (14)	2.9958 (12)	173.8 (12)
N14—H14B⋯N2	0.884 (15)	2.209 (15)	3.0836 (12)	170.1 (13)
N4—H4B⋯N1 *^i^*	0.884 (14)	2.355 (14)	3.2242 (12)	167.7 (12)
N4—H4B⋯N2 *^i^*	0.884 (14)	2.659 (14)	3.5055 (12)	160.6 (11)
N14—H14A⋯N11 *^ii^*	0.900 (15)	2.403 (15)	3.2741 (13)	163.0 (12)

Symmetry codes: (*i*) x − 1, y, z; (*ii*) x + 1, y, z.

**Table 3:** Characteristic normal modes and their PED contribution to the skeleton in the studied triazolopyridine derivatives (the calculated values for the monomer are listed in parentheses).

	TPa-NH_2_	6MTPb	7MTPHb
ν_as_(Φ)	(1324) 1340-1339	(1326) 1323-1322	(1275) 1275
ν_s_(Φ)	(767) 770-766	(760) 776-762	(678) 699
γ(Φ)—wagging	(426) 430-425	(440) 440-439	(550) 521-515
τ(Φ)—wagging	(188) 192-191	(250) 273-268	(187) 209-204

**Table 4:** Calculated singlet and triplet electron levels of the TPa-NH_2_ molecule.

Electron Levels	Wavelengths [nm]		Wavenumber [cm^−1^]		Oscillator Strength	
	Monomer	Dimer	Monomer	Dimer	Monomer	Dimer
singlets						
(1)	326.35	347.81	30,642	28,751	0.0409	0.0003
(2)	270.76	347.03	36,933	28,816	0.0019	0.0801
(3)	249.50	326.74	40,080	30,605	0.0297	0.0009
(4)	224.49	326.40	44,545	30,637	0.0310	0.0001
(5)	216.58	261.22	46,172	38,282	0.0418	0.0000
(6)	211.79	260.64	47,216	38,367	0.2960	0.0047
(7)	204.21	253.45	48,969	39,455	0.1038	0.0005
(8)	200.29	253.29	49,928	39,480	0.0124	0.1183
(9)	195.66	242.08	51,109	41,309	0.0027	0.0004
(10)	188.01	241.91	53,189	41,338	0.0553	0.0001
triplets						
(1)	478.55	504.98	20,896	19,803	0.0000	0.0000
(2)	319.77	504.82	31,272	19,809	0.0000	0.0000
(3)	301.54	328.58	33,163	30,434	0.0000	0.0000
(4)	291.26	328.32	34,333	30,458	0.0000	0.0000
(5)	239.07	327.45	41,829	30,539	0.0000	0.0000
(6)	235.13	327.26	42,530	30,557	0.0000	0.0000
(7)	231.32	307.60	43,230	32,510	0.0000	0.0000
(8)	230.06	307.34	43,467	32,537	0.0000	0.0000
(9)	226.85	278.28	44,082	35,935	0.0000	0.0000
(10)	211.93	277.75	47,185	36,004	0.0000	0.0000

## Data Availability

The data presented in this study are available on request from the corresponding author.

## Supplementary Materials

Table S1: Crystallographic data and final refinement parameters for TPa-NH_2_; Table S2: The Mulliken atomic charges determined for the studied derivative; Table S3: The stabilization energies E(2) associated with the hyper conjugative interaction within the molecule; Table S4: Experimental and scaled wavenumbers (in cm^−^^1^) of the vibrational spectra for TPa-NH_2_ monomer and dimer; checkCIF/PLATON report.
