# Supplementary material for: The Structural and Optical Properties of 1,2,4-Triazolo[4,3-a]pyridine-3-amine
Source: Molecules. 2022 Jan 22;27(3):721. doi: 10.3390/molecules27030721 (PMC8838196; doi:10.3390/molecules27030721)

checkCIF/PLATON report

Structure factors have been supplied for datablock(s) 123AP_100

THIS REPORT IS FOR GUIDANCE ONLY. IF USED AS PART OF A REVIEW PROCEDURE FOR PUBLICATION, IT SHOULD NOT REPLACE THE EXPERTISE OF AN EXPERIENCED CRYSTALLOGRAPHIC REFEREE.

No syntax errors found.   CIF dictionary   Interpreting this report

Datablock: 123AP_100

Bond precision:	C-C = 0.0014 A		Wavelength=0.71073
Cell:	a=5.5666(3)	b=12.6649(6)	c=16.8190(8)
	alpha=90	beta=99.434(7)	gamma=90
Temperature:	100 K		

	Calculated	Reported
Volume	1169.71(10)	1169.71(10)
Space group	P 21/n	P 21/n
Hall group	-P 2yn	-P 2yn
Moiety formula	C6 H6 N4	C6 H6 N4
Sum formula	C6 H6 N4	C6 H6 N4
Mr	134.15	134.15
Dx,g cm-3	1.523	1.524
Z	8	8
Mu (mm-1)	0.103	0.103
F000	560.0	560.0
F000'	560.14	
h,k,lmax	7,16,21	7,16,21
Nref	2682	2655
Tmin,Tmax	0.973,0.982	0.981,1.000
Tmin'	0.973	

Correction method= # Reported T Limits: Tmin=0.981 Tmax=1.000
AbsCorr = MULTI-SCAN

Data completeness= 0.990   Theta(max)= 27.499

R(reflections)= 0.0315(2387)   wR2(reflections)= 0.0807(2655)

S = 1.020   Npar= 197

The following ALERTS were generated. Each ALERT has the format
test-name_ALERT_alert-type_alert-level.
Click on the hyperlinks for more details of the test.

Alert level C

```
PLAT230_ALERT_2_C Hirshfeld Test Diff for    N4       --C1       .        5.5 s.u.
PLAT480_ALERT_4_C Long H...A H-Bond Reported H4B      ..N2       .       2.66 Ang.
PLAT918_ALERT_3_C Reflection(s) with I(obs) much Smaller I(calc) .          1 Check
```

Alert level G

```
PLAT910_ALERT_3_G Missing # of FCF Reflection(s) Below Theta(Min).          2 Note
PLAT912_ALERT_4_G Missing # of FCF Reflections Above STh/L=  0.600         25 Note
PLAT961_ALERT_5_G Dataset Contains no Negative Intensities .......     Please Check
PLAT965_ALERT_2_G The SHELXL WEIGHT Optimisation has not Converged     Please Check
PLAT978_ALERT_2_G Number C-C Bonds with Positive Residual Density.         10 Info
```

0 **ALERT level A** = Most likely a serious problem - resolve or explain
0 **ALERT level B** = A potentially serious problem, consider carefully
3 **ALERT level C** = Check. Ensure it is not caused by an omission or oversight
5 **ALERT level G** = General information/check it is not something unexpected

0 ALERT type 1 CIF construction/syntax error, inconsistent or missing data
3 ALERT type 2 Indicator that the structure model may be wrong or deficient
2 ALERT type 3 Indicator that the structure quality may be low
2 ALERT type 4 Improvement, methodology, query or suggestion
1 ALERT type 5 Informative message, check

It is advisable to attempt to resolve as many as possible of the alerts in all categories. Often the minor alerts point to easily fixed oversights, errors and omissions in your CIF or refinement strategy, so attention to these fine details can be worthwhile. In order to resolve some of the more serious problems it may be necessary to carry out additional measurements or structure refinements. However, the purpose of your study may justify the reported deviations and the more serious of these should normally be commented upon in the discussion or experimental section of a paper or in the "special_details" fields of the CIF. checkCIF was carefully designed to identify outliers and unusual parameters, but every test has its limitations and alerts that are not important in a particular case may appear. Conversely, the absence of alerts does not guarantee there are no aspects of the results needing attention. It is up to the individual to critically assess their own results and, if necessary, seek expert advice.

Publication of your CIF in IUCr journals

A basic structural check has been run on your CIF. These basic checks will be run on all CIFs submitted for publication in IUCr journals (*Acta Crystallographica*, *Journal of Applied Crystallography*, *Journal of Synchrotron Radiation*); however, if you intend to submit to *Acta Crystallographica Section C* or *E* or *IUCrData*, you should make sure that full publication checks are run on the final version of your CIF prior to submission.

Publication of your CIF in other journals

Please refer to the *Notes for Authors* of the relevant journal for any special instructions relating to CIF submission.

PLATON version of 13/07/2021; check.def file version of 13/07/2021

Datablock 123AP_100 - ellipsoid plot

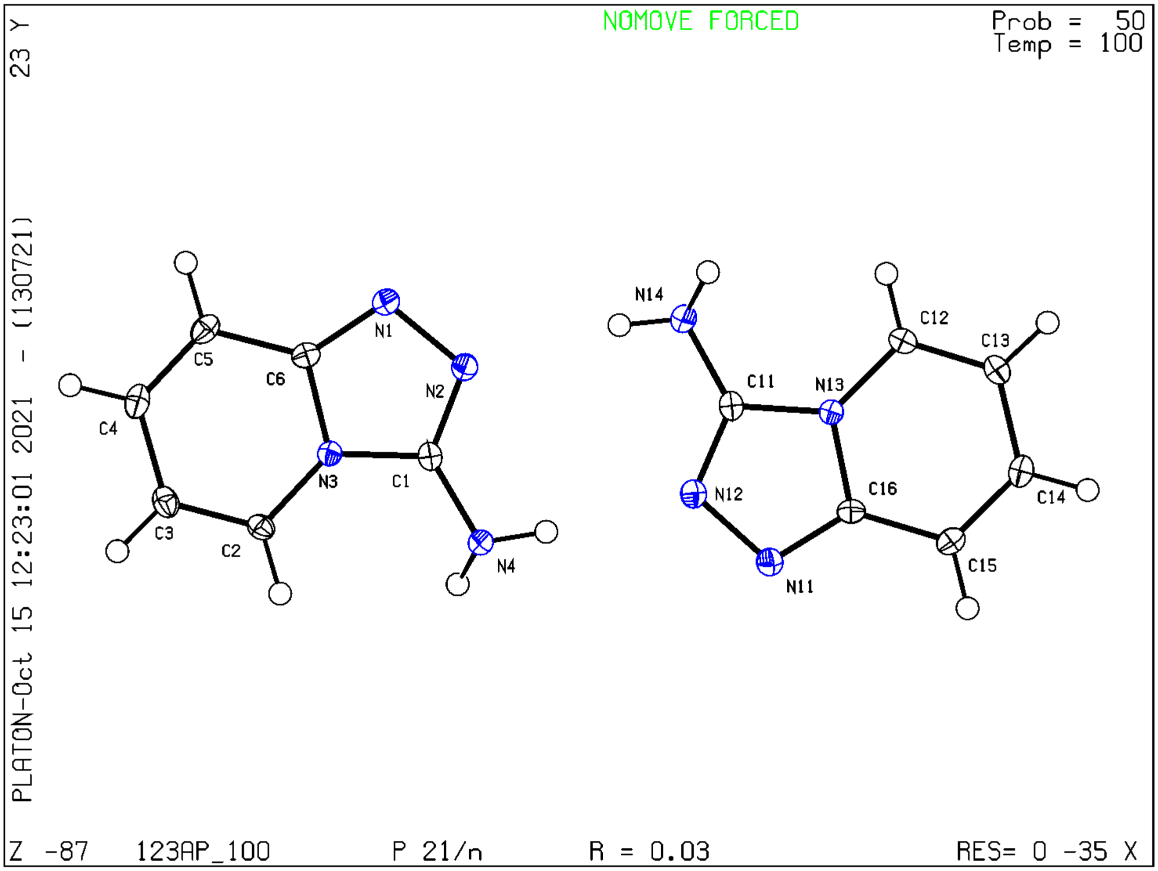

Supplement: Supplementary file 1 [file molecules-27-00721-s001.zip › checkcif_123AP_100.pdf]
