# Supplementary material for: The Structural and Optical Properties of 1,2,4-Triazolo[4,3-a]pyridine-3-amine
Source: Molecules. 2022 Jan 22;27(3):721. doi: 10.3390/molecules27030721 (PMC8838196; doi:10.3390/molecules27030721)
Supplement: Supplementary file 1 [file molecules-27-00721-s001.zip › table S1.pdf]

Table S1. Crystallographic data and final refinement parameters for TPa-NH_2.

Formula	$C_6H_6N_4$
Molecular weight	134.15
Temp. (K)	100 (2)
Crystal system	Monoclinic
Space group	*P 2₁/n*
a (Å)	5.5666(3)
b (Å)	12.6649(6)
c (Å)	16.8190(8)
β (°)	99.434(8)
V (Å^3)	1169.71(10)
Z	8
F(000)	560
D_{cal} (g·cm^{-1})	1.524
θ range(°)	3.410 - 29.193
μ (mm^{-1})	0.103
Crystal size (mm)	0.267 - 0.232 - 0.176
T_{min} / T_{max}	0.9812 / 1.000
Total / unique / obs refls	14316 / 2655 / 2387
R_{int}	0.0156
R [$F^2>2\sigma(F^2)$][a]	0.0315
wR [F^2 all refls][a]	0.0807
S	1.020
$\Delta\rho_{max}$, $\Delta\rho_{min}$ (eÅ^{-3})	+0.217, -0.233

[a] $R=\Sigma ||F_o|-|F_c||/\Sigma F_o$, $wR=\{\Sigma [w(F_o^2-F_c^2)^2]/\Sigma wF_o^4\}^{1/2}$; $w^{-1}=\sigma^2(F_o^2) + (aP)^2 + bP$ where $P = (F_o^2 + 2F_c^2)/3$. The *a* and *b* parameters are 0.0403 and 0.4085, respectively.
