# Supplementary material for: The Structural and Optical Properties of 1,2,4-Triazolo[4,3-a]pyridine-3-amine
Source: Molecules. 2022 Jan 22;27(3):721. doi: 10.3390/molecules27030721 (PMC8838196; doi:10.3390/molecules27030721)
Supplement: Supplementary file 1 [file molecules-27-00721-s001.zip › table S2.pdf]

Table S2. The Mulliken atomic charges determined for the studied derivative.

	**NBO**		**Mulliken**	
	**monomer**	**dimer**	**monomer**	**dimer**
N1	-0.312	-0.312	-0.279	-0.289
N2	-0.320	-0.390	-0.281	-0.322
N3	-0.399	-0.402	-0.243	-0.247
N4	-0.828	-0.834	-0.406	-0.405
C1	0.533	0.567	0.420	0.477
C2	0.028	0.30	0.118	0.118
C3	-0.243	-0.245	-0.183	-0.183
C4	-0.196	-0.195	0.044	0.045
C5	-0.202	-0.203	-0.163	-0.166
C6	0.334	0.333	0.372	0.368
H1	0.210	0.209	0.084	0.086
H2	0.214	0.214	0.063	0.063
H3	0.207	0.206	0.068	0.067
H4	0.224	0.233	0.067	0.065
H5	0.365	0.365	0.152	0.141
H6	0.386	0.433	0.166	0.188
