# Supplementary material for: The Structural and Optical Properties of 1,2,4-Triazolo[4,3-a]pyridine-3-amine
Source: Molecules. 2022 Jan 22;27(3):721. doi: 10.3390/molecules27030721 (PMC8838196; doi:10.3390/molecules27030721)
Supplement: Supplementary file 1 [file molecules-27-00721-s001.zip › table S3.pdf]

Table S3. The stabilization energies E(2) associated with the hyper conjugative interaction within the molecule.

**Donor NBO (i)**	**Acceptor NBO (j)**	**E(2) [kcal/mol]**	**E(j)-E(i)**
σ(N1-N2)	σ*(C5-C6)	6.17	1.30
σ(N1-N2)	σ*(N4-C1)	6.57	1.18
σ(N2-C1)	σ*(N3-C2)	4.51	1.27
π(N2-C1)	π*(N1-C6)	12.04	0.33
σ(N1-C6)	σ*(C5-C6)	4.22	1.36
σ(N1-C6)	σ*(N3-C2)	3.27	1.27
π(N1-C6)	π*(N2-C1)	14.79	0.31
π(N1-C6)	π*(C4-C5)	12.35	0.33
σ(C5-H4)	σ*(N3-C6)	5.12	0.93
σ(C5-H4)	σ*(C3-C4)	4.40	1.04
σ(C5-C6)	σ*(N1-C6)	4.47	1.26
σ(C4-C5)	σ*(N1-C6)	4.09	1.29
π(C4-C5)	π*(N1-C6)	21.77	0.29
π(C4-C5)	π*(C2-C3)	16.42	0.29
σ(C6-N3)	σ*(N4-C1)	5.18	1.19
σ(C3-H2)	σ*(C4-C5)	3.28	1.13
σ(C3-H2)	σ*(N3-C2)	5.59	0.96
σ(C3-C4)	σ*(C2-H1)	3.19	1.11
σ(C2-C3)	σ*(N3-C1)	4.55	1.18
π(C2-C3)	π*(C4-C5)	14.85	0.32
σ(C4-H3)	σ*(C5-C6)	4.69	1.04
σ(C4-H3)	σ*(C2-C3)	3.56	1.12
σ(N3-C1)	σ*(C5-C6)	3.22	1.34
σ(C2-H1)	σ*(N3-C6)	5.16	0.96
σ(C2-H1)	σ*(C3-C4)	3.80	1.07
σ(N4-H5)	π*(N2-C1)	4.08	0.65
σ(N4-H6)	σ*(N3-C1)	6.28	1.08
n(N2)	σ*(N1-C6)	6.22	0.93
n(N2)	σ*(N3-C1)	9.03	0.81
n(N1)	σ*(N2-C1)	6.47	0.93
n(N1)	σ*(N3-C6)	9.19	0.77
n(N3)	π*(N2-C1)	42.80	0.29
n(N3)	π*(N1-C6)	38.61	0.29
n(N3)	π*(C2-C3)	33.67	0.29
n(N4)	σ*(N2-C1)	5.00	0.90
n(N4)	π*(N2-C1)	16.82	0.34
π*(N1-C6)	π*(C4-C5)	191.52	0.01
π*(C2-C3)	π*(C4-C5)	109.69	0.01
