# Supplementary material for: The Structural and Optical Properties of 1,2,4-Triazolo[4,3-a]pyridine-3-amine
Source: Molecules. 2022 Jan 22;27(3):721. doi: 10.3390/molecules27030721 (PMC8838196; doi:10.3390/molecules27030721)
Supplement: Supplementary file 1 [file molecules-27-00721-s001.zip › table S4.pdf]

Table S4. Experimental and scaled wavenumbers (in cm^{-1}) of the vibrational spectra for TPa-NH_2 monomer and dimer.

**No.**	**Calculated wavenumbers (IR absorbance, RS intensity in %)**		**Experimental wavenumbers**		**PED contributions**
			**IR**	**RS**	
	**monomer**	**dimer**	**A_u modes**	**A_g modes**	
ν_1	3431 (38, 16)	3404, 3403	3330 s		100 $\nu_{as}(NH_2)$
ν_2	3342 (6, 55)		3293 s		100 $\nu_s(NH_2)$
		3126, 3096	3153 s		$\nu(NH_B)$: ν(N-H···N) HB vibration
ν_3	3083 (4, 44)	3082, 3082	3088 sh	3094 m	98 $\nu(CH)_P$
ν_4	3073 (3, 36)	3072, 3071	3074 s	3079 m	98 $\nu(CH)_P$
ν_5	3063 (3, 32)	3063, 3063			98 $\nu(CH)_P$
ν_6	3045 (6, 24)	3044, 3044	3032 s		98 $\nu(CH)_P$
ν_7	1650 (43, 25)	1675, 1675		1648 w	$\delta(NH_2)$<br>68 $\nu(\phi_P)$ + 12 $\delta(CH)_P$
ν_8	1632 (54, 45)	1649, 1648	1633 s	1633 m	86 $\delta(NH_2)$
ν_9	1543 (66, 80)	1563, 1552	1562 s	1554 vs	30 $\nu(\phi_P)$ + 20 $\nu(\phi)_T$ + 18 $\nu(C\text{-}NH_2)$ + 17 $\delta(\phi_T)$
ν_{10}	1533 (33, 84)	1537, 1537		1527 m	33 $\nu(\phi_T)$ + 30 $\nu(\phi_P)$ + 12 $\delta(\phi_T)$
ν_{11}	1511 (40, 99)	1515, 1515	1507 s	1509 vs	58 $\nu(\phi_P)$ + 12 $\nu(C\text{-}NH_2)$
ν_{12}	1459 (10, 14)	1460, 1459	1453	1469 vw<br>1455 s	52 $\delta(CH)_P$ + 28 $\nu(\phi_P)$
ν_{13}	1419 (39, 16)	1428, 1426	1420 s	1423 m	38 $\nu(\phi_T)$ + 33 $\delta(C\text{-}NH_2)$ + 13 $\delta(NH_2)$<br>in plane-bending δ(N−H···N) HB vibration
ν_{14}	1371 (10, 2)	1380, 1379	1370 s	1372 m	52 $\delta(CH)_P$ + 33 $\nu(\phi_T)$
ν_{15}	1324 (37, 100)	1331, 1331	1339 s	1340 s	51 $\nu(\phi_P)$ + 30 $\nu(\phi_T)$ = $\nu_{as}(\Phi)$
ν_{16}	1282 (3, 4)	1284, 1284	1282 m	1285 vw	48 $\delta(CH)_P$ + 12 $\nu(\phi_P)$ + 11 $\nu(\phi_T)$
ν_{17}	1181 (0, 24)	1195, 1192	1189 w	1193 w<br>1183 w	61 $\delta(CH)_P$ + 15 $\nu(\phi_P)$
ν_{18}	1148 (3, 16)	1162, 1153	1148 m	1148 m	59 $\delta(CH)_P$ + 19 $\delta(NH_2)$
ν_{19}	1139 (4, 42)	1145, 1144	1130 w	1139 m	41 $\delta(NH_2)$ + 15 $\delta(CH)_P$ + 14 $\nu(\phi_T)$
ν_{20}	1094 (14, 13)	1102, 1100	1105 m	1106 w	17 $\nu(C\text{-}NH_2)$ + 15 $\delta(\phi_T)$ + 13 $\delta(CH)_P$ + 10 $\nu(\phi_P)$<br>+ 10 $\nu(\phi_T)$
ν_{21}	1055 (29, 72)	1062, 1058	1026 m	1024 m	87 $\nu(\phi_T)$
ν_{22}	995 (4, 42)	994, 994	997 w	999 m	63 $\delta(CH)_P$ + 20 $\nu(\phi_P)$
ν_{23}	978 (0, 2)	976, 976	967 w	972 w<br>967 w	52 $\gamma(CH)_P$ + 23 $\nu(\phi_P)$
ν_{24}	934 (0, 3)	932, 932	947 w	948 w	100 $\gamma(CH)_P$
ν_{25}	915 (2, 43)	915, 915	920 m	919 m	42 $\delta(\phi_T)$ + 27 $\delta(\phi_P)$
		884, 870			$\gamma(NH_2)$ out of plane-bending γ(N−H···N) HB vibration
ν_{26}	837 (98, 6)		848 m		93 $\gamma(CH)_P$
ν_{27}	826 (100, 14)	828, 827	828 m	824 w	62 $\gamma(NH_2)$ + 31 $\gamma(C\text{-}NH_2)$
ν_{28}	767 (17, 45)	778, 777	766 m	770 s	29 $\nu(\phi_P)$ + 15 $\delta(\phi_T)$ + 10 $\nu(C\text{-}NH_2)$ = $\nu_s(\Phi)$

ν_{29}	750 (26, 9)	754, 750			$48\ \gamma(CH)_P + 16\ \gamma(\phi_P\backslash\phi_T) + 12\ \gamma(\phi_P)$
ν_{30}	744 (3, 36)	747, 747		747 w	$31\ \nu(C\text{-}NH_2) + 25\ \nu(\phi_T) + 19\ \delta(\phi_P)$
ν_{31}	735 (54, 3)	736, 735	732 s		$34\ \gamma(CH)_P + 16\ \gamma(\phi_P\backslash\phi_T) + 14\ \gamma(\phi_P)$
ν_{32}	676 (12, 2)	692, 687	682 m<br>658 m		$79\ \gamma(\phi_T) + 14\ \gamma(\phi_P)$
ν_{33}	597 (4, 52)	601, 597	597 m	597 m	$45\ \delta(\phi_P) + 35\ \nu(\phi_P) + 18\ \delta(\phi_T)$
ν_{34}	583 (4, 1)	584, 583		570 w	$70\ \gamma(\phi_P) + 12\ \gamma(\phi_T)$
ν_{35}	557 (5, 38)	562, 557	554 m	553 m	$53\ \delta(\phi_P) + 22\ \nu(\phi_P) + 16\ \delta(\phi_T)$
ν_{36}	505 (10, 11)	523, 516	518 w	516 m	$42\ \delta(C\text{-}NH_2) + 38\ \delta(\phi_P) + 19\ \delta(\phi_T)$
ν_{37}	426 (8, 10)	427, 425<br>384, 371	456 m<br>425 m<br>351 w	430 m<br>350 vw	$67\ \gamma(\phi_P) + 15\ \gamma(\phi_P\backslash\phi_T)$ = $\gamma(\Phi)$ wagging
ν_{38}	332 (6, 5)	340, 339			$55\ \gamma(C\text{-}NH_2) + 39\ \gamma(\phi_P)$
ν_{39}	254 (6, 85)	291, 285	295 m<br>286 w	296 m<br>283 s	$40\ \delta(C\text{-}NH_2) + 23\ \delta(\phi_P) + 10\ \nu(\phi_P)$
ν_{40}	225 (29, 67)		228 vw<br>221 vw	221 w	$88\ \gamma(NH_2) + 10\ \gamma(\phi_P\backslash\phi_T)$
ν_{41}	188 (6, 52)	203, 196	192 vw	191 w	$81\ \gamma(\phi_P\backslash\phi_T) + 18\ \gamma(NH_2)$ = $\tau(\Phi)$ wagging
ν_{42}	158 (3, 63)	162, 161		130 w	$34\ \gamma(C\text{-}NH_2) + 20\ \gamma(\phi_P) + 17\ \gamma(\phi_P\backslash\phi_T) + 10\ \gamma(\phi_T)$
		104	115 w	111 s	$\nu(NH)\cdots N$: $\nu(N\text{-}H\cdots N)$ HB vibration
		76, 58			$\delta(NH_BN)$
		48, 32, 16			$\gamma(NH_BN)$

Calculated values were scaled using the factors: 0.955 for ν_1–ν_6 and 0.985 for ν_7–ν_{42} normal modes. Abbreviations: ϕ_P - pyridine ring; ϕ_T - triazole ring; Φ – ($\phi_P + \phi_T$) ; ν - stretching; δ - in-plane bending; γ and τ - out-of-plane bending; H_B – hydrogen atom engaged in the HB
